# The combination of graphene oxide and preservatives can further improve the preservation of cut flowers

**DOI:** 10.3389/fpls.2023.1121436

**Published:** 2023-03-14

**Authors:** Yuyang Wu, Yuerui Wang, Siyuan Wang, Xiaotan Fan, Yuran Liu, Runxuan Zhao, Haijiang Hou, Yixin Zha, Jinhua Zou

**Affiliations:** Tianjin Key Laboratory of Animal and Plant Resistance, College of Life Sciences, Tianjin Normal University, Tianjin, China

**Keywords:** cut rose, preservative, graphene oxide, nanomaterials, anti-oxidation

## Abstract

It is reported that the use of nanomaterials can extend the vase life of fresh-cut flowers. Graphene oxide (GO) is one of these nanomaterials that aid in promoting water absorption and antioxidation during the preservation of fresh-cut flowers. In this investigation, the three mainstream brands of preservatives commercially available on the market (“*Chrysal*,” “*Floralife*,” and “*Long Life*”) in combination with low concentrations of GO (0.15 mg/L) were used to preserve fresh-cut roses. The results showed that the three brands of preservatives had different degrees of freshness retention. Compared to the preservatives used alone, the combination of low concentrations of GO with the preservatives, especially in the L+GO group (with 0.15 mg/L GO added in the preservative solution of “*Long life*”), further improved the preservation of cut flowers. L+GO group showed less level of antioxidant enzyme activities, lower ROS accumulation and cell death rate, and higher relative fresh weight than the other groups, implying a better antioxidant and water balance abilities. GO attached to the xylem duct of flower stem, and reduced the blockage of xylem vessels by bacteria, which were determined by SEM (scanning electron microscopy) and FTIR (Fourier transform infrared) analysis. XPS (X-ray photoenergy spectra) analysis results proved that GO could enter the interior of flower stem through xylem duct, and when combined with “*Long Life*,” the anti-oxidation protection ability of GO was enhanced, thus delaying ageing, and greatly extending the vase life of fresh-cut flowers. The study provides new insights into cut flower preservation using GO.

## Introduction

Fresh-cut flowers have become increasingly popular as people’s living standards have improved. Cut roses, the world’s most popular ornamental plant, have a vase life of only a few days when their stems are separated from the parent plants (Mazrou et al., 2022). How to extend the viewing time of cut roses effectively is a major concern for scholars. Currently, popular preservatives can extend the viewing time of cut roses by 4–5 d (Ahmad et al., 2014), and ultimately, the main cause of cut flower death is microbial blooms that clog the stems (Fang et al., 2021; Spricigo et al., 2021) and reactive oxygen species (ROS) accumulation (Hassan et al., 2020; Zeng et al., 2023). Although the vase life of cut flowers is extended, the existing preservatives are still worth improving.

Nanomaterials are widely used in many fields, such as biomedicine, mechanical engineering, and electronics, due to their special chemical properties. The potential of nanomaterials for agricultural applications is gradually being discovered, for example, Xu et al. (2017) discovered that the nanocomposite film prepared with graphene oxide (GO), titanium dioxide (TiO_2_), and chitosan at a 1:20:4 ratio had a significant bacterial inhibition effect without any cytotoxicity to plant and mammalian somatic cells. Spanò et al. (2019) demonstrated a significant reduction of cadmium (Cd) ions in the roots and leaves of rice under TiO_2_ nanoparticle treatment. Babu and Tingirikari (2023) reviewed that various nanomaterials were applied in food industry by exhibiting antimicrobial and biotherapeutic properties, enhancing the shelf-life, and maintaining food quality. Atefepour et al. (2021) demonstrated 15 mg/L of silver nanoparticles combined with 6% sucrose increased the vase life of cut Gerbera (*Gerbera jamesonii*) flowers. Nanomaterials have shown positive effects in the preservation of fresh-cut flowers due to their excellent water absorption and antibacterial capacity, thus extending the vase life of fresh-cut flowers (Spricigo et al., 2021).

GO, a graphene derivative and carbon-based nanomaterial, has oxygen-containing functional groups in its structure that make it highly hydrophilic and surface active (McCoy et al., 2019). Recent studies on GO as an antimicrobial agent have shown that low concentrations of GO can extend the vase life of cut flowers (He et al., 2018). GO can directly increase the activities of antioxidant enzymes and the contents of some plant hormones (Zhao et al., 2022) and can also effectively improve the water retention capacity of plants (Zhao et al., 2020). However, there are few research results in the area of the preservation of fresh-cut flowers by using nanomaterials, and there are no studies on the fresh-keeping effect of GO combined with preservatives on fresh-cut flowers.

The preservation effects of the three most popular preservatives on the market were compared, and the fresh-keeping effect of low concentrations of GO combined with preservatives on fresh-cut roses was also explored in this investigation. The parameters such as relative fresh weight, antioxidant enzyme activities, and vase life were measured, and the characteristics of the stem incisions of cut flowers were determined using microscopical observation and scanning electron microscopy (SEM). Fourier transform infrared (FTIR) spectra and X-ray photoenergy spectra (XPS) were also used to characterize the stem ends of the cut flowers. The above investigations allow the exploration of the potential of GO to improve the preservative effect and provide a feasible solution for preservative enhancement, as well as explore the role of GO in extending the vase life of cut flowers.

## Materials and methods

### Experimental group settings

The experiments were carried out using three of the most popular preservatives on the market, namely, “*Chrysal* (Chrysal Clear consumer bottle, Chrysal, Holland),” “*Floralife* (Floralife 200, Floralife, Chicago, US),” and “*Long Life* (Longlife Sachet, Gadot Agro Co., Israel).” According to the optimum concentration provided by the manufacturers of the different preservatives, 0.5 L culture solution of each brand of preservative was prepared and named C, F, and L, respectively. GO reagent was purchased from XFNANO Materials Tech Co., Ltd., China. Three combination groups (namely C+GO, F+GO, and L+GO) were prepared by dissolving GO (0.075 mg of GO in each group) in the same three solutions as the preservative groups. In addition, 0.5 L of tap water was used in the control group (abbreviated as CK), and 0.075 mg of GO was dissolved in 0.5 L of tap water in the GO group (abbreviated as GO). Cut roses (*Rosa chinensis* cv. *carola*) were purchased from a local flower market in Tianjin, China, and immediately delivered to the laboratory. Cut roses with a similar growth status and openness were cut vertically to 0.25 m at the ends of their stems, and all their leaves were removed. The stems of the treated cut roses were rinsed using distilled water and placed in the eight treatment solutions for incubation at 23–26°C, under a relative humidity of 18–38%. On the sixth day of the experiment, the culture solution was replenished to 0.5 L. Three replicates were set for each group.

### Vase life and relative fresh weight of cut roses

The vase life of cut flowers is an important indicator for the viewing of fresh-cut flowers (Fatima et al., 2022). Vase life was calculated as the number of cultivation days from the time that the cut roses were placed in the culture solution (Day 0) to the day that severe neck bowing or extensive shedding and wilting of petals occurred. The fresh weight of the cut roses was measured during the observation period. In addition, photographs of the same bundle of cut roses of every treatment group were taken with a digital camera on Day 3, 6, 9, and 12.

The fresh weight (FW) of the cut roses was measured during the observation period to calculate the relative fresh weight (RFW) using the formula:


$$\text{RFW }\left(\text{g}\right)=\text{ F}{\text{W}}_{\text{d}}–\text{ F}{\text{W}}_{0}$$

Where FW_d_ is the fresh weight of the cut roses (g) on d = Day 3, 6, 9, and 12, and FW_0_ is the weight of the cut roses (g) on Day 0.

### Analysis of the activities of antioxidant enzymes

The effect of protective enzymes can be detected and assessed by the changes in the activities of superoxide dismutase (SOD), peroxidase (POD), and catalase (CAT) (Kong et al., 2021; Zeng et al., 2023). The petals of the cut roses were selected randomly for antioxidant enzyme studies on Day 3, 6, 9, and 12 after starting the experiment. POD activity was determined using the guaiacol method to measure the increase in absorbance at 470 nm according to the method of Zou et al. (2017), and the POD activity unit was U/g·min. According to the method of Wu et al. (2017), SOD activity was determined and defined as the amount of enzyme that caused a 50% inhibition of nitro-blue-tetrazolium (NBT) reduction at 560 nm, expressed as U/mg. CAT activity was measured by monitoring the absorbance decrease at 240 nm due to the degradation of H_2_O_2_, and the unit was U/g (Wu et al., 2017).

### *In situ* detection of reactive oxygen species and evaluation of cell death

The outer petals of the cut roses were collected on Day 3, 6, 9, and 12 after starting the experiment. For visualization and measurement of ROS, *in situ* H_2_O_2_ detection was carried out by 3,3’-diaminobenzidine (DAB) staining (Zou et al., 2022), while *in situ* O_2_^-^ detection was carried out by using NBT staining (Zou et al., 2022).

The outer petals of the cut roses from each treatment group were stained using Evans blue stain on Day 3, 6, 9, and 12, and the extent of cell death was assessed by the size of the stained area in the outer petals from each group (Zhang et al., 2021). The percentage of the stained area of petals on Day 12 was calculated using Image J software (NIH, Bethesda, MD, USA).

### Characterization of the stem ends

After 6 days of treatment, small sections of 1–2 cm from the bottom of the flower stems of each treatment were observed by using a stereomicroscope. Some other stem segments were first frozen in liquid nitrogen for half an hour and then freeze-dried with a lyophilizer (Telstar, Lyoqlest-85, Spain). The stem segments were sputter-coated (Emitech K550X, UK) and then observed using SEM (FEI Nova NanoSEM 230, USA) to characterize the morphology of the incised xylem vessels of the samples from each group. Afterward, the surfaces of the freeze-dried stem segments, which contacted the treatment solutions directly, from the C and C+GO groups were analyzed using an FTIR spectroscope (Nicolet iS50, USA) to characterize the special peak of the GO attached on the surface of the stem ends, with a wavenumber range from 500 to 4000 cm^−1^. The surfaces of the freeze-dried stem segments, which contacted the treatment solutions indirectly, from the L and L+GO groups were examined using XPS (Axis-Ultra-Dld, U.K.) to analyze the redox state of the GO attached to the inner surface of the xylem vessels.

## Results and discussion

### Effects of different treatments on the vase life and fresh weight of the cut roses

**Figure 1:**
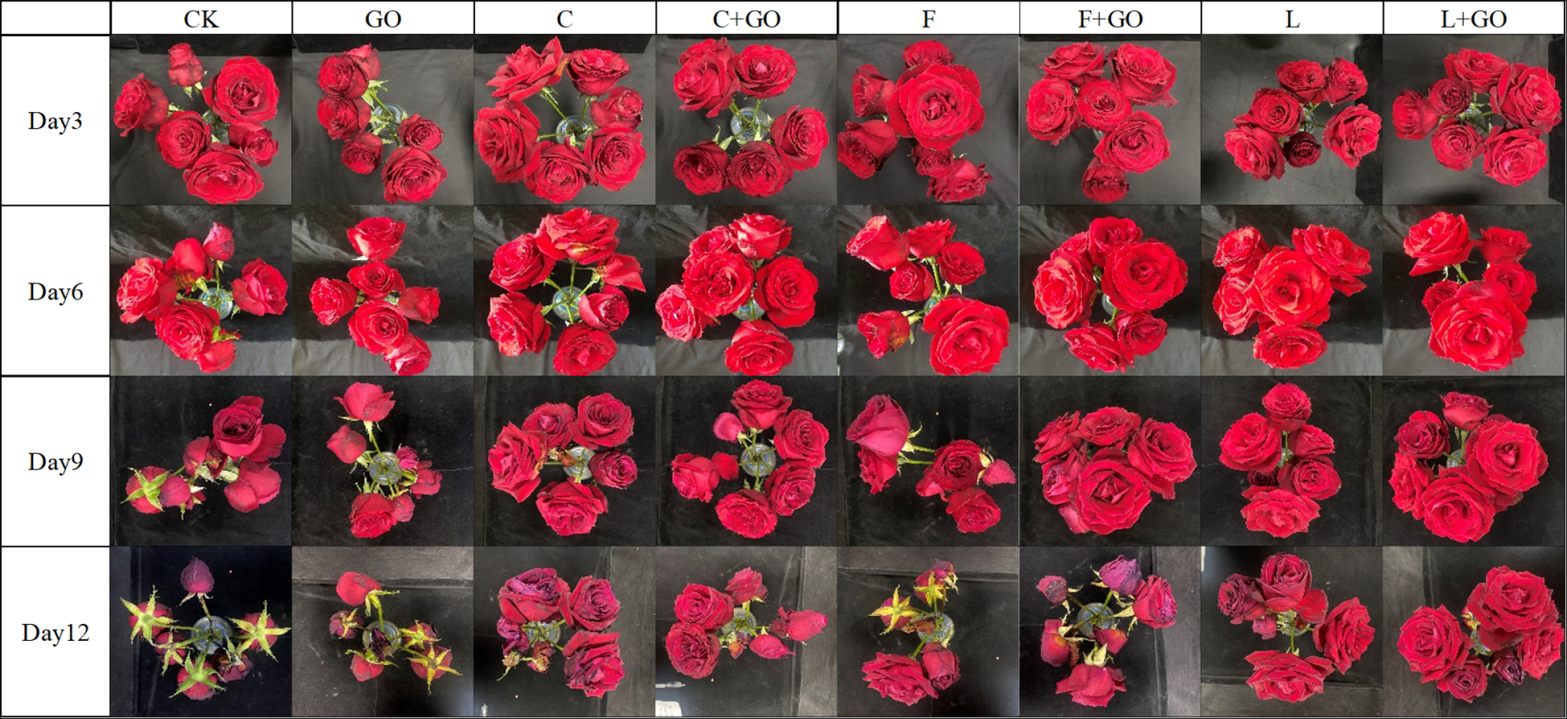
Top view of the cut rose flowers in different treatments.

Photographs of the cut roses were taken on Day 3, 6, 9, and 12 to directly observe the effect of the different treatments on the freshness of the cut roses. After 12 days of treatment, the L and L+GO groups had the best fresh-keeping effect and retained a high ornamental value, followed by the C+GO group, while the roses in the other groups wilted (**Figure 1**).

**Figure 2:**
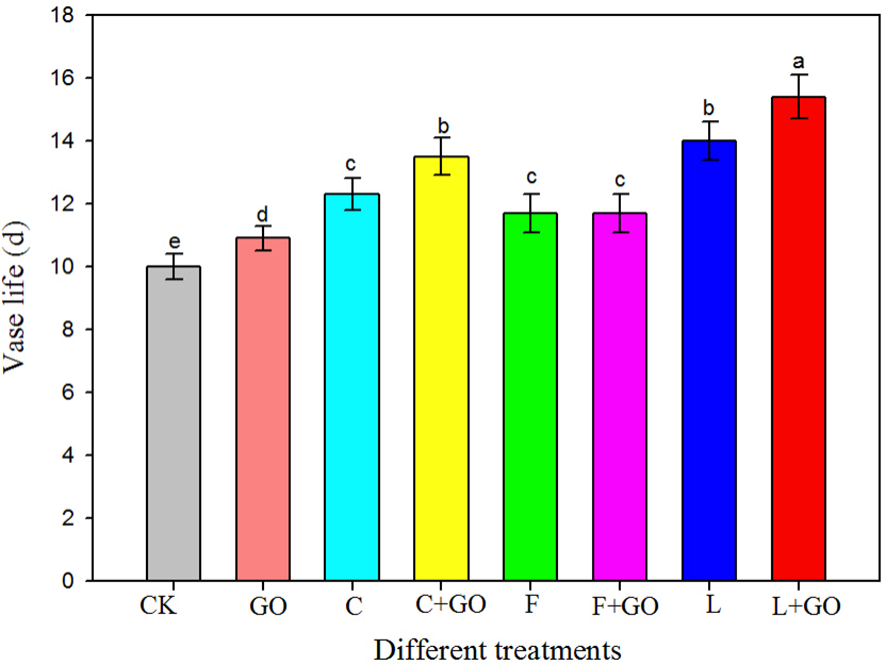
Vase life of the fresh cut roses in different treatment groups. Different lowercase letters indicate significant difference among different groups at p< 0.05.

**Figure 2** shows that the vase life of the roses in the L+GO, C+GO, and L groups was significantly longer than that of the roses in the other groups. Compared to CK (control), the three groups with preservatives alone extended the vase life of the cut roses, while the roses in the L group had a longer vase life compared to those in the C and F groups. Therefore, the three preservatives were all helpful for prolonging the vase life of the cut roses. Compared with the CK group, the average vase life of the roses in the L group was 4 days longer, far exceeding that of the roses treated with the other two preservatives, indicating that “*Long Life*” had the most obvious preservative effect. However, among the three combination groups with GO and preservatives, except for the F+GO group, the roses in the L+GO and C+GO groups exhibited a longer vase life than that in the L and C groups, respectively. A low concentration of GO combined with “*Long Life*” and “*Chrysal*” enhanced their preservative effects, while a low concentration of GO combined with “*Floralife*” did not show a better preservative effect than “*Floralife*.” In general, low concentrations of GO extended the vase life of the cut roses in the investigation, consistent with the results of He et al. (2018) and Thakur et al. (2022).

**Figure 3:**
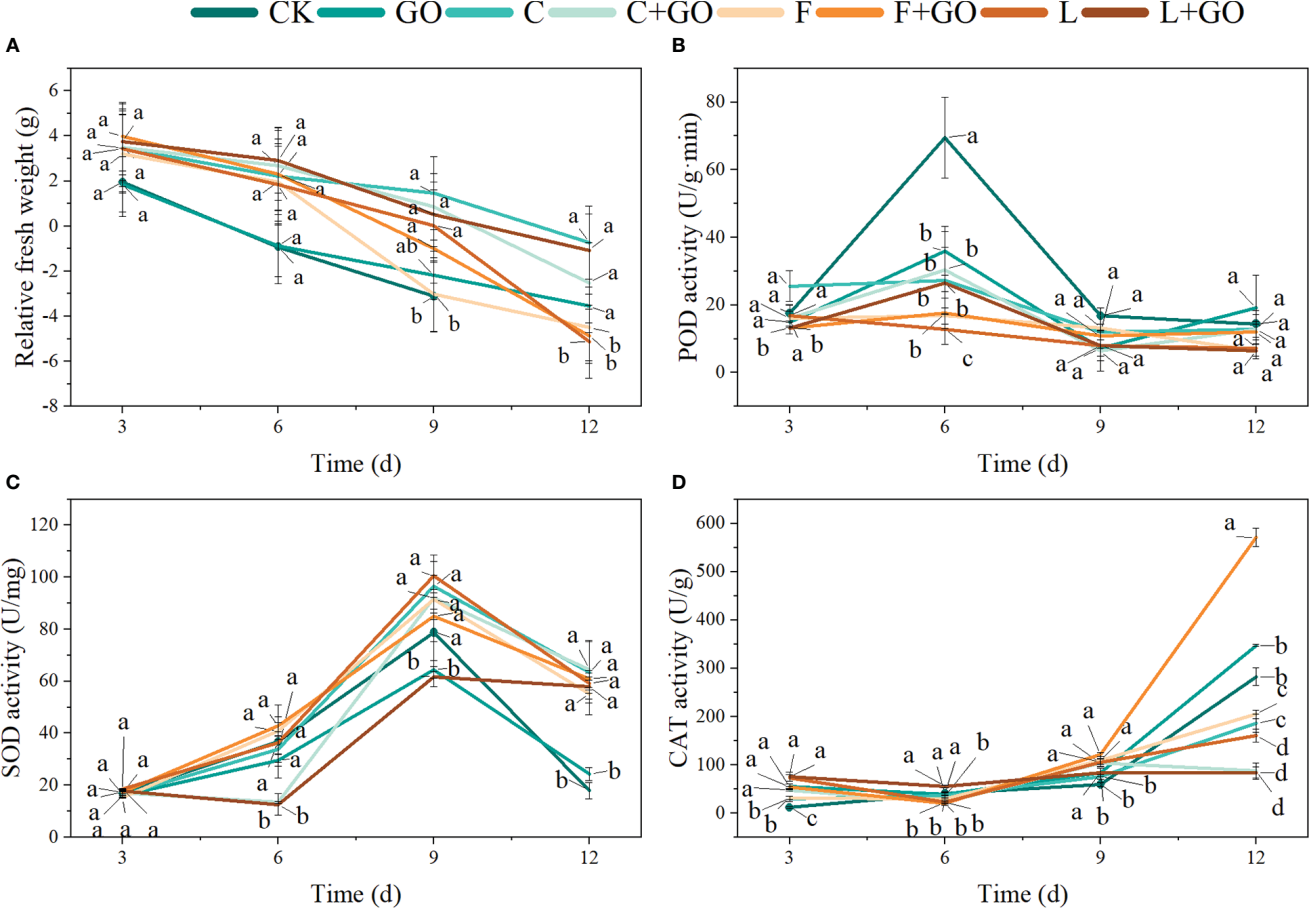
Effects of different treatments on the relative fresh weight, and the activities of superoxide dismutase (SOD), peroxidase (POD), and catalase (CAT) in the petals of the cut rose flowers. **(A)** Relative fresh weight. **(B)** POD activity. **(C)** SOD activity. **(D)** CAT activity. Different lowercase letters indicate significant difference among different groups at p< 0.05.

From **Figure 3A**, it can be seen that the RFW of the roses in all the eight groups showed a decreasing trend from Day 3, 6, and 9 to Day 12, indicating that the cut flowers were in a state of gradual water loss during the incubation, which could be a key cause of cut flowers’ mortality. However, each group exhibited a different rate of water loss. On Day 3, the RFW of CK and GO was lower than that of the groups with preservatives only, and the decrease of RFW in the CK and GO groups was more pronounced on Day 6. On Day 9, the RFW of the F and GO groups was markedly lower than that of the other groups (*P*<0.05). When the treatment time reached 12 days, all the cut flowers in the CK group had died, and by this time, the RFW of the L, F and F+GO groups was significantly lower than that of the other groups (*P*<0.05). Taken together, the above results showed that “*Chrysal*” and “*Long Life*” had a higher water retention capacity than tap water. The three combination groups with GO and preservatives showed a decline in FW similar to that in the other groups at the beginning of the experiment and a slower decline than that in the other groups in the middle and end of the experimental period, suggesting that the use of GO in combination with preservatives did improve the water retention capacity of the cut flowers compared with the groups with the preservative only. Among them, the L+GO group showed the most pronounced water-retention effect, which indicated that a better water imbalance occurred in the L+GO group during the preservation of the fresh-cut flowers (Ahmad et al., 2011; Hassan et al., 2020; Zhao et al., 2020).

### Effects of different treatments on the activities of antioxidant enzymes in cut roses

Current studies have shown that ROS accumulate largely during the aging process of plants (Zeng et al., 2023). The excessive accumulation of ROS induces the stress response of the plant antioxidant defense system, leading to changes in the activities of antioxidant enzymes, among which SOD, POD, and CAT play an important role in reducing ROS production; so, the activities of SOD, POD, and CAT are important indicators for assessing the senescence degree of cut flowers (He et al., 2018; Hassan et al., 2020).

As shown in **Figure 3B**, except in the L group, the POD activities tended to increase first, then decrease, and then increase slowly. On Day 3, the POD activities of F+GO and L+GO were significantly lower than those of the other groups (*P*<0.05), and on Day 6, the POD activities of the CK group were significantly higher than those of the other groups (*P*<0.05). On Day 9 and 12, the POD activities of L, C+GO, and L+GO were lower than those of the other groups.

Except for the L+GO and C+GO groups, the SOD activities of the other groups exhibited a trend of increasing first and then decreasing. On Day 3, the SOD activities of the L+GO and C+GO groups were significantly lower than those of the other groups (*P*<0.05), while the SOD activities of the L+GO and C+GO groups both showed a substantial increase on Day 9. The SOD activities of the CK and GO groups suffered a substantial decline on Day 12 compared to those on Day 9 and were lower than those of the other groups (**Figure 3C**).

The CAT activities of all groups increased continuously during the whole experiment. On Day 12, the F+GO group had the highest CAT activity, followed by the GO and CK groups, while the L, C+GO, and L+GO groups had the lowest CAT activities, which were significantly different from that of the other groups (*P*<0.05) (**Figure 3D**).

The trend of the three antioxidant enzymes during the overall experimental period was similar to the results of Thakur et al. (2022). All three antioxidant enzymes in the L+GO group were at a low level during the whole cultivation except SOD activities on Day 12, with minimal changes from Day 6 to 12. Combining the actual growing conditions of the L+GO group, the average vase life, and the effects of the antioxidant enzymes on plant senescence, it can be deduced that the three antioxidant enzymes under L+GO treatment were increased to a higher level after 12 days, with the peak occurring later than those in the other groups. This occurred for the POD activity in group L and the CAT activity in group C+GO. The growth status and average vase life of these two groups (L and C+GO) were lower only than that of the L+GO group, confirming the previous speculation that the peak antioxidant enzyme activity appeared later, and L+GO was more effective than L and C+GO in preserving freshness. The reason for this situation might be because the cut flowers in the L, L+GO, and C+GO groups accumulated less ROS and had lower senescence at the beginning of the cultivation, and the antioxidant enzyme activities showed a lower level than those of the other groups (Hassan et al., 2020). SOD activities of the CK and GO groups on Day 12 showed the least levels, which was due to the flowers of the CK and GO groups had wilted and lost the antioxidant abilities on Day 12 (**Figure 1**).

### Effects of different treatments on ROS accumulation in cut rose petals

#### Diaminobenzidine staining result

**Figure 4:**
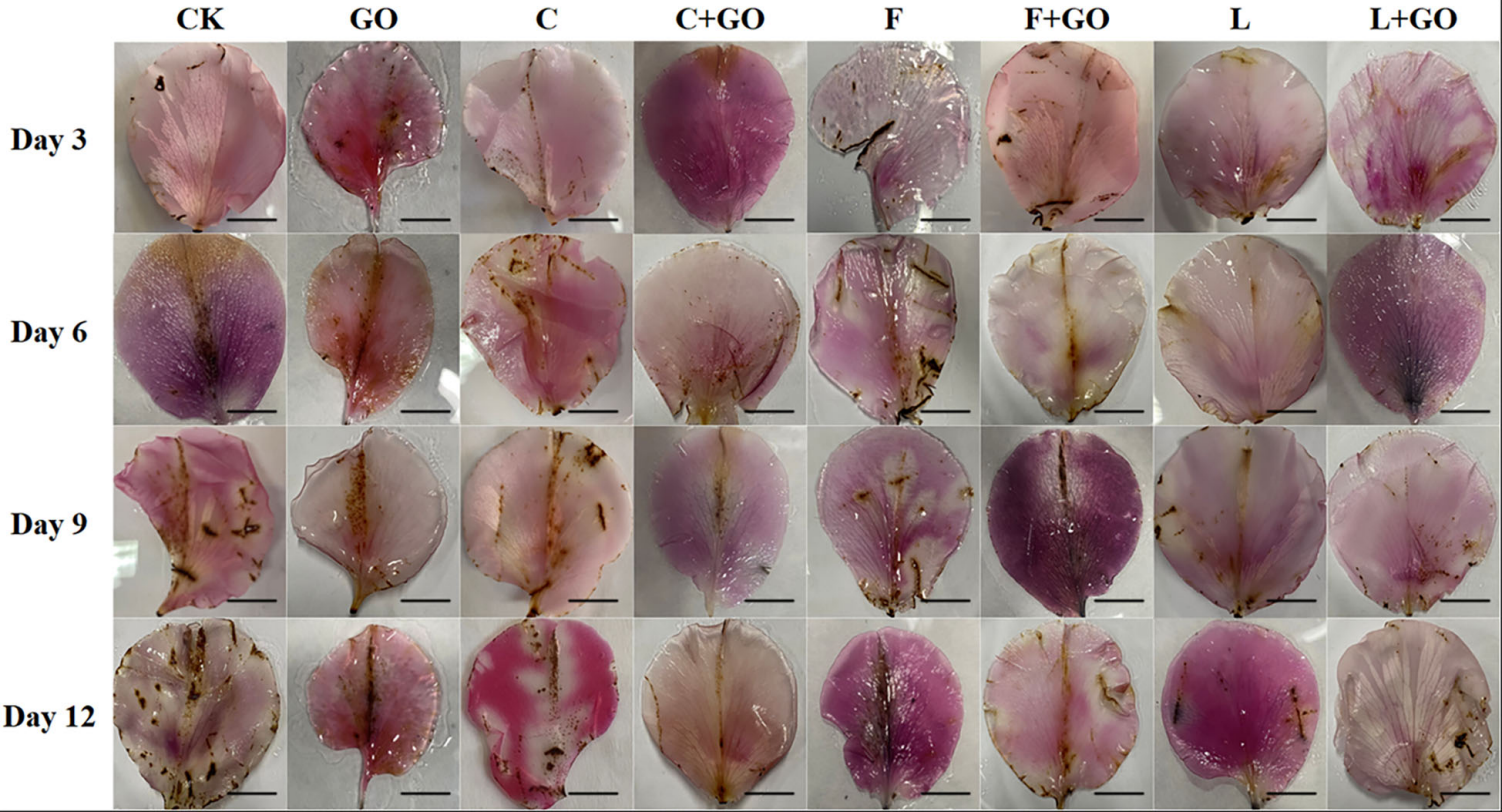
DAB staining results of the petals of the cut rose flowers after 12 d of treatment. Scale bar: 1 cm.

**Figure 5:**
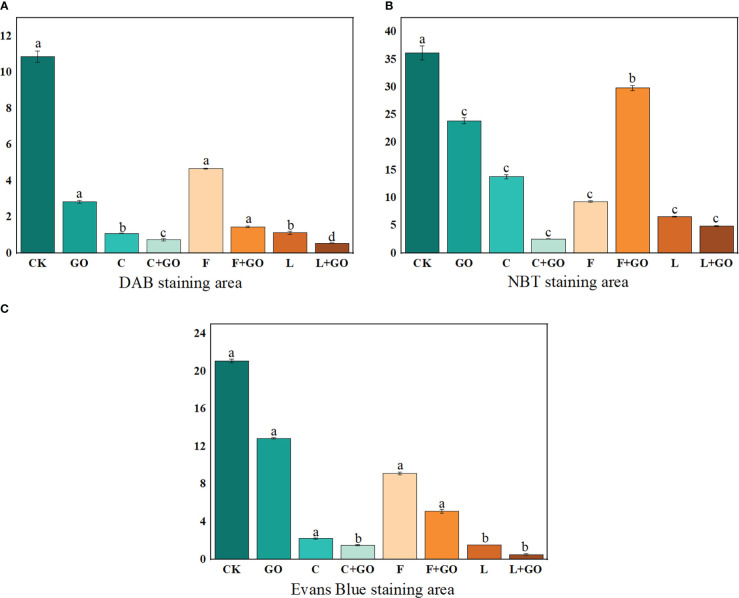
Staining area ratio of the cut rose petals after 12d of treatment. **(A)** DAB staining. **(B)** NBT staining. **(C)** Evans Blue staining.

DAB staining showed the *in situ* distribution of H_2_O_2_ in the petals of cut roses (Zou et al., 2022). As shown in **Figure 4**, with the increase in the treatment time, the brown area of each treatment group became increasingly large, and the brown color deepened gradually. By calculating the percentage of the stained area of petals on Day 12 using Image J software (NIH, Bethesda, MD, USA), the area of DAB accumulation in the L, L+GO, and C+GO groups was 1.59%, 1.31%, and 1.06%, respectively (**Figure 5A**), much smaller than that in the other treatment groups, indicating that the H_2_O_2_ accumulation in the petals of these three groups was lower than that of the other groups. Among all eight groups, the CK group had the darkest brown color with the largest percentage of area, reaching 14.52% (**Figure 5A**), implying that the CK group accumulated the most amount of H_2_O_2_. By comparing the three preservative groups without GO addition with the three preservative groups with GO added, it could be found that the brown area in the first three groups was smaller than that in the latter three groups.

#### Nitro-blue tetrazolium staining result

**Figure 6:**
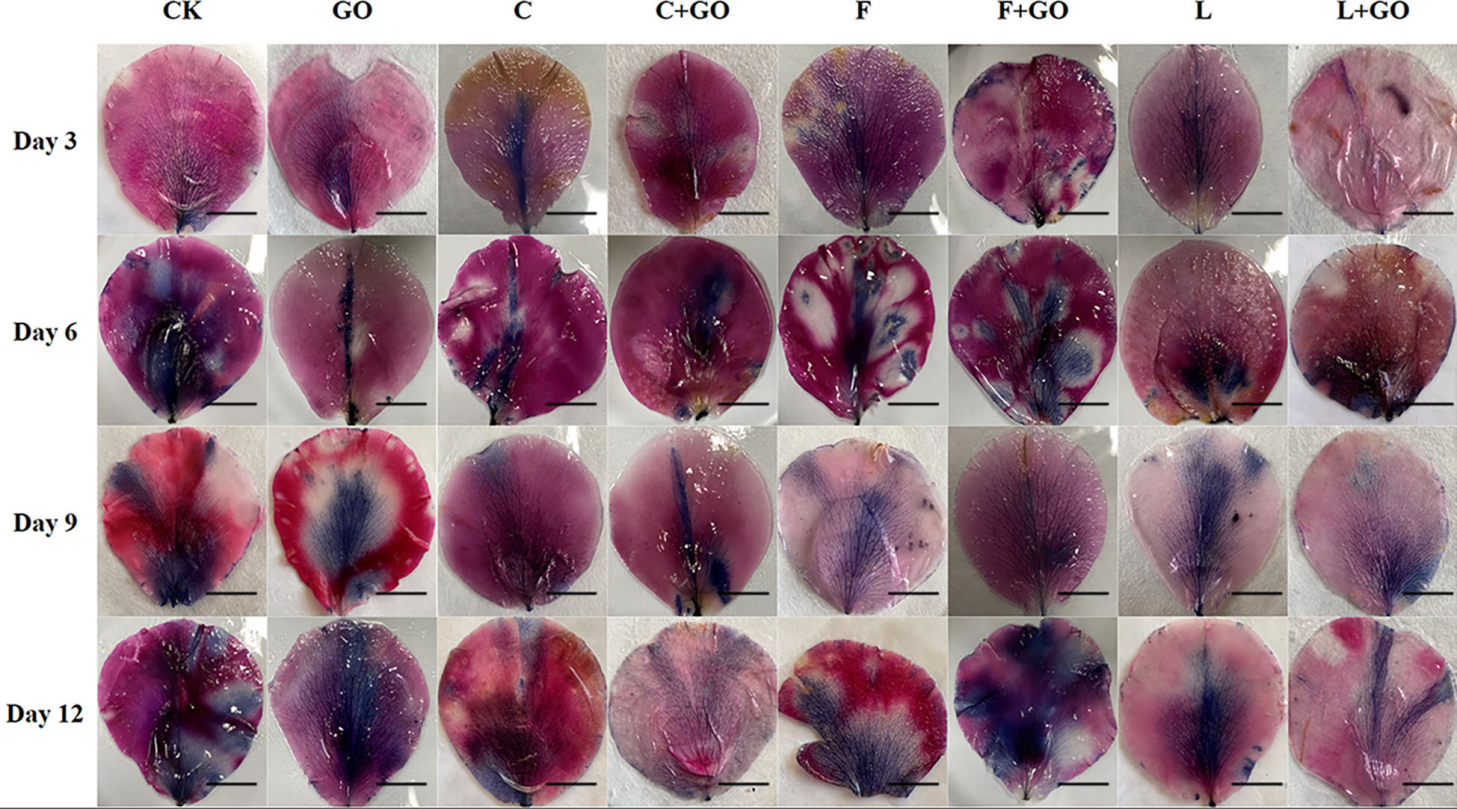
NBT staining results of the petals of the cut rose flowers after 12 d of treatment. Scale bar: 1 cm.

NBT staining showed the *in situ* distribution of O_2_^-^, in the petals of cut roses (Zou et al., 2022). It can be seen from **Figure 6** that the area of the NBT-stained part of all treatment groups gradually expanded and the color gradually deepened with the increase in the treatment time. The staining results on Day 12 were obtained by using Image J software (**Figure 5B**). It was found that the L+GO and C+GO groups had the smallest blue area and the lightest color, with NBT staining area 3.29% and 7.13%, respectively, indicating that the petals of these two groups had the lowest accumulation of O_2_^-^. In addition, the blue area in the three groups with GO was smaller than that in the corresponding three groups without GO.

The dysregulation of water uptake in cut flowers during incubation is an important factor in the accumulation of ROS (In et al., 2017). A low concentration of GO was attached to the cut flower stems, which contributed to relieving the imbalance of water uptake in cut flowers, thus reducing the large accumulation of ROS in the flowers. The staining areas of the petals in the L, C+GO, and L+GO groups for both NBT staining and DAB staining were smaller than those of the other groups, fully indicating that these three groups, especially the L+GO group, were more effective in scavenging ROS from the petals than the other groups. The above results reflected that the combination of GO with preservatives could reduce the production of ROS in the petals of the cut rose flowers.

### Effects of different treatments on the cell death rate of the cut rose petals

**Figure 7:**
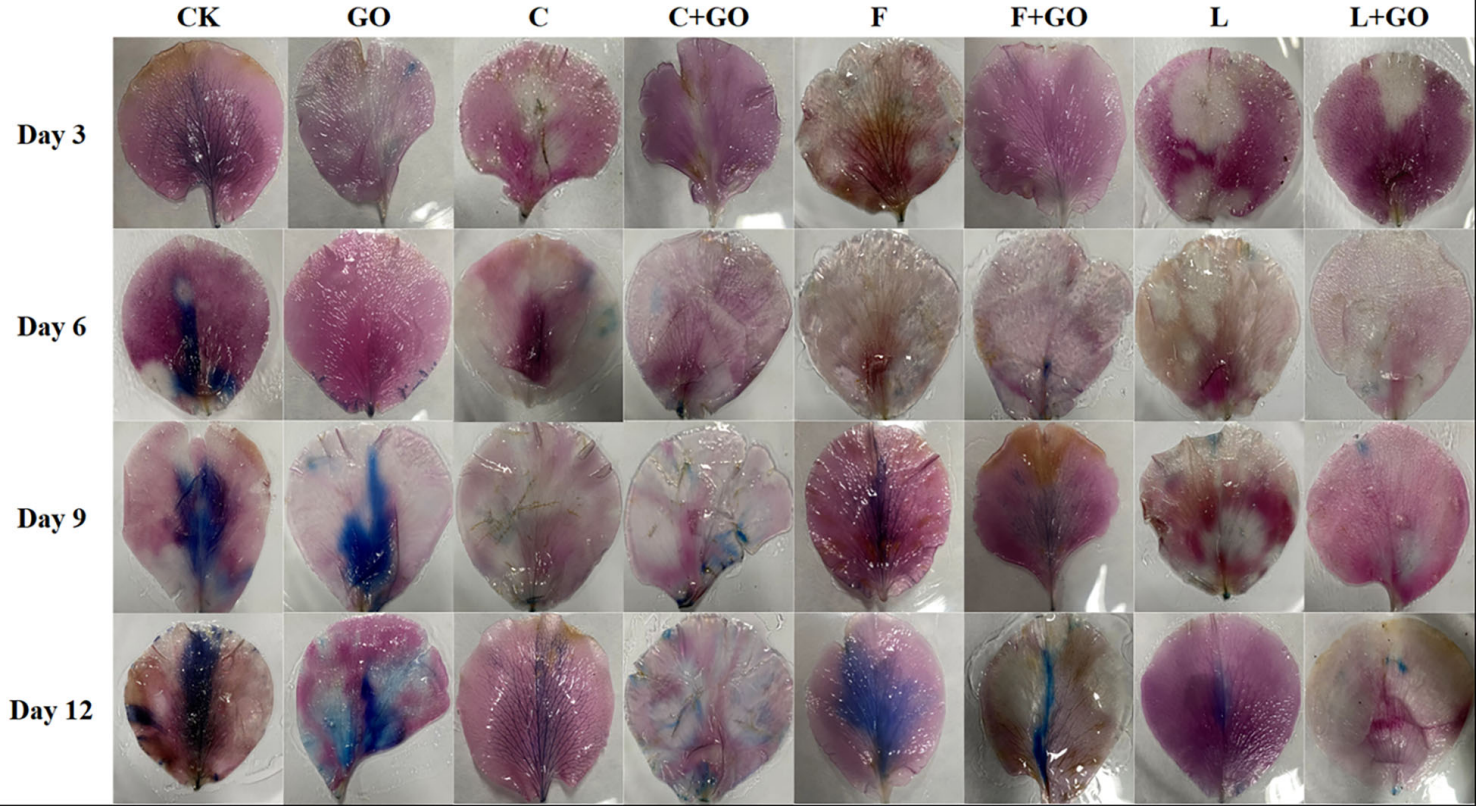
Evans Blue staining of the petals of the cut rose flowers after 12 d of treatment. Scale bar: 1 cm.

Evans blue can access dead cells, but cannot penetrate the cell membrane of living cells, so Evans blue staining usually serves as a marker of cell death (Zhang et al., 2021; Zou et al., 2022). Evans blue staining results are shown in **Figure 7**. It can be seen that on Day 3 and 6, a large blue area appeared in the CK group, while there was no obvious blue color in the other groups. On Day 9, the blue color of the CK group deepened and the area expanded. At the same time, a large blue area appeared in the GO group, a small blue area appeared in the F group, and the remaining groups had only a few punctate distributions. On Day 12, except in the L+GO group in which the blue area was still not obvious, the blue areas of the other groups were distributed in patches. Based on the calculated results using Image J, it was found that the CK group had the largest percentage of blue area, 30.32%, and was the darkest, followed by the F (14.60%), GO (12.06%), and F+GO (4.28%) groups (**Figure 5C**). All four groups with GO were found to have a smaller blue area and a lighter color than the four groups without GO, which demonstrated that the combined use of GO and preservatives reduced the cell death rate of the cut rose petals.

### Characteristics of the stem ends of the cut roses

#### Stereomicroscope characterization

**Figure 8:**
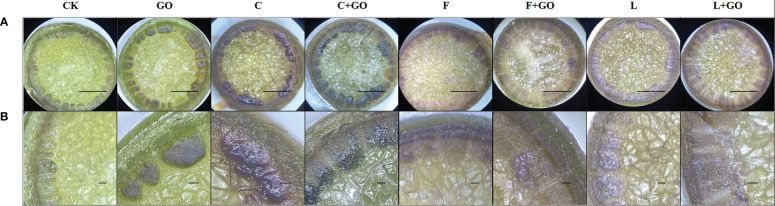
Micrographs of the stem incisions of the cut rose flowers after 6 d of treatment observed using a stereomicroscope. Scale bars: 1 mm in **(A)**, 0.1 mm in **(B)**.

In the cross-section of the rose stems, the epidermis, cortex, and vascular column were clearly observed, and the center of the rose stem were occupied by a large area of pith tissues. As can be seen in **Figure 8**, rings of dark brown attachments were found on the surface of the vascular tissues at ends of the flower stems, which were formed by the attachment of bacteria and their secretions or GO. It could be observed that in the region of xylem vessels, the groups with GO were darker than the other groups without GO, which was due to the black GO nano-particles themselves attached in the region of xylem vessels. He et al. (2018) also reported the above similar phenomenon on flower stem cuttings under a low concentration of GO. The characteristics of GO attachments at stem ends were analyzed subsequently using FTIR and XPS.

#### Scanning electron microscope characterization

**Figure 9:**
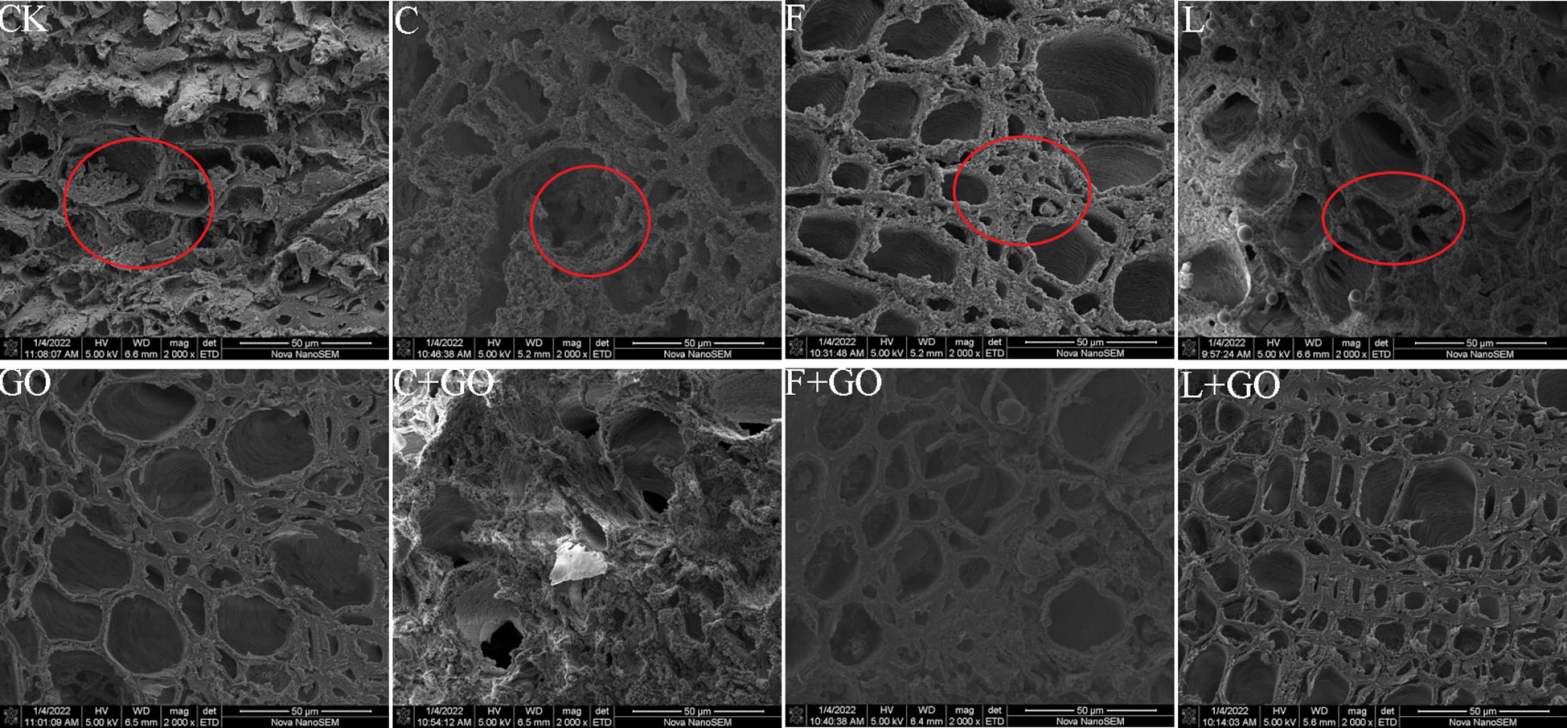
Micrographs of the stem incisions of the cut rose flowers after 6 d of treatment observed using scanning electron microscopy. Scale bar: 50 μm.

GO addition has an antibacterial effect, which increases as the concentration increases; so, GO can speed-up water absorption (Sun et al., 2015). **Figure 8** shows the cross-sectional morphology of the xylem of the fresh-cut roses immersed in the treatment solutions for 6 days. The xylem is composed of xylem vessels, fibers, and xylem parenchyma cells, while the xylem vessels are mainly used for water transport. It was found that the xylem vessels in all four groups without GO addition were blocked by bacteria and their secretions to different degrees (red circles in **Figure 9**). When compared with the CK and GO groups, it was obvious that the CK group showed serious blockage, while the GO group with showed less blockage of xylem vessels, proving that the low concentration of GO did act as a bacterial inhibitor during the preservation of cut flowers. Microorganisms multiplied in the culture during the keeping process of cut flowers, causing blockage of the xylem vessels of the stem cuttings (In et al., 2017), while GO addition might be a good solution to this problem, allowing GO to extend the vase life of the cut flowers.

### FTIR analysis

**Figure 10:**
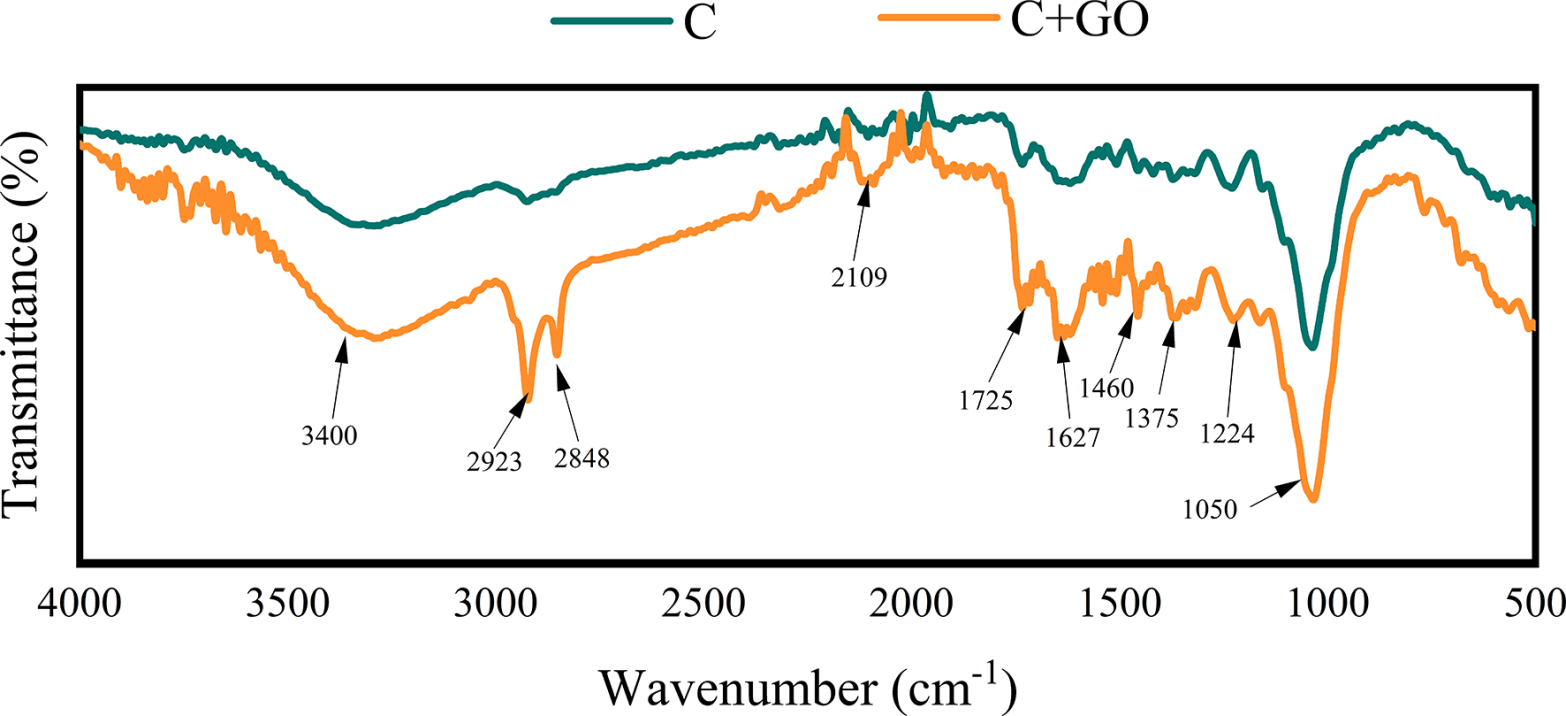
Characteristics of the stem incisions from the C and C+GO groups examined by Fourier transform infrared spectroscopy.

FTIR spectra were used to analyze the surface of the stem ends from the C and C+GO groups, and it was shown that the characteristic peaks of C+GO at 3400 cm^-1^ were generated by the stretching vibration of -OH (Rajkumar and Vijayakumar, 2017), while the peak at 1375 cm^-1^ was generated by the bending vibration on the -OH surface. The characteristic peak at 1725 cm^-1^ was generated by the contraction vibration of C=O in -COOH (Sudesh et al., 2013), and the characteristic peak at 1627 cm^-1^ was generated by the stretching vibration of C=C (Wazalwar and Raichur, 2021). The peak at 1050 cm^-1^ was due to the contraction vibration of C-O (Yang et al., 2012). From **Figure 10**, it can be seen that GO was indeed attached to the stem ends of the C+GO group, but there was no GO present in the stem ends of the C group because of the absence of the characteristic peak at 1725 cm^-1^ in the stem ends of this group. Furthermore, the absorption peaks of -CH_2_ at 2923 cm^-1^, 2848 cm^-1^, and 1460 cm^-1^, the absorption peak of the R-C≡C-H stretching vibration at 2109 cm^-1^, and the characteristic peak generated by C-O-C at 1224 cm^-1^ were present in the stem ends of the C+GO group, but they were absent in the stem ends of the C group (**Figure 10**), which also proved that GO attached to the stem ends of the combination groups of GO and preservatives due to the transpiration pull. Consequently, GO could play an antibacterial role in reducing the blockage of xylem vessels (In et al., 2017; Xu et al., 2017; He et al., 2018).

### XPS analysis

**Figure 11:**
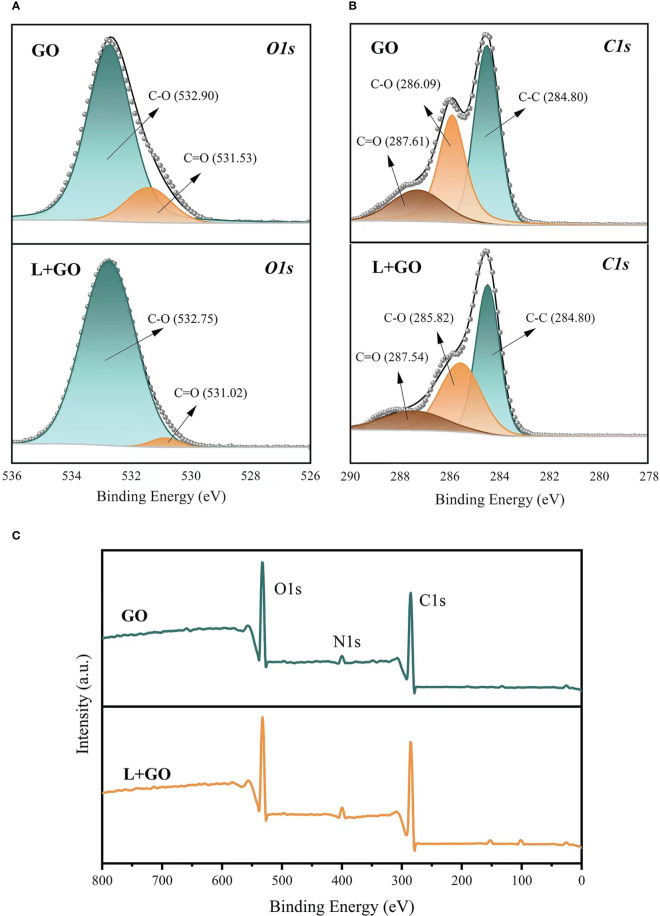
Characteristics of the stem incisions from the GO and L+GO groups examined by X-ray photoenergy spectra (XPS). **(A)** C1s XPS spectra. **(B)** O1s XPS spectra. **(C)** XPS wide survey.

In the previous experiments, the combination of preservatives and GO demonstrated a powerful preservation effect (He et al., 2018). In this investigation, a more visible fresh-keeping effect was found in the L+GO group than in the C+GO and F+GO groups. To further investigate the degree of oxidation-reduction of the GO attached to the xylem vessels, the L+GO and GO groups were subjected to XPS analysis to analyze whether the antioxidation protection capability of GO could be enhanced when combined with preservatives. Higher amounts of C1 and O1, as constituents of GO, were detected in the xylem vessels from both the GO and L+GO groups (**Figure 11A**). The small amount of N element present at the same time in xylem vessels might be due to the introduction of a few nitrogen-containing groups into GO by light, forming the corresponding nitrides (Wang et al., 2018). By calculating the peak area ratios of C1s (C-C, C-O, C=O) for the two groups (GO and L+GO), it was found that the C-C peak area of the L+GO group was 45.21%, while the C-C peak area of the GO group decreased to 43.36%. The C-O and C=O peak areas of the L+GO group were 35.36% and 19.44%, respectively, and the C-O and C=O peak areas of the GO group increased to 36.65% and 20.00%, respectively, fully demonstrating that the GO in the L+GO group was in a state of less oxidation than that in the GO group (**Figures 11A, B**). The lower oxidation status indicated that, when combined with the most effective preservative, “*Long Life*,” the anti-oxidation protection ability of GO was enhanced, further reducing the accumulation of ROS in cut flowers, thus delaying the senescence of the cut flowers. DAB staining results (**Figure 4**) and NBT staining results (**Figure 6**) showed that the lowest amount of ROS accumulation was found in the petals of the L+GO group among all the eight groups, and **Figures 7**, **5C** also showed that less cell death was found in the L+GO group than in the other groups. All of the above staining results supported the conclusion of less oxidation in the petals of the L+GO group. Akhavan and Ghaderi (2012) also stated that the bactericidal effect of GO was correlated with its degree of oxidation- reduction. It can be seen from **Figures 11A, B** that the C1s and O1s peaks in the L+GO group had lower binding energies, which may be related to the absorption of substances from “*Long Life*” by GO, which explained why GO combined with preservatives played stronger bactericidal roles and had a stronger fresh-keeping effect than when GO was used alone.

The cut flowers in this study were cultured in a normal indoor environment without any light or temperature protection for the cut flower solution, thus providing a reliable basis for the home culture of cut flowers. It is worth noting that the stems of the fresh cut flowers showed varying degrees of bending during cultivation, most noticeably in the CK and GO groups, while the combination of preservatives and GO seemed to improve the bending appearance slightly, which was presumably related to the entry of GO into the stems of cut flowers and the improved water uptake and balance, which need further experimental proof. More detailed concentration gradients of GO in combination with preservatives could be used in future experiments to explore the optimum ratio of the two. In addition, what is the effect of GO on the scent of cut roses, which can be further explored.

## Conclusions

Different brands of preservatives had different fresh-keeping effects on cut flowers. Of these brands of preservatives, “*Long Life*” was the most effective, and low concentrations of GO combined with preservatives further enhanced their ability to retain freshness. “*Long Life*” and “*Chrysal*” were the two best preservatives in combination with GO, with a longer ornamental life. *Long Life*” combined with GO also showed less level of antioxidant enzyme activities, lower ROS accumulation and cell death rate, and higher relative fresh weight than the other groups, implying a better antioxidant and water balance capabilities. SEM observation showed that GO attached to the xylem duct of cut flowers and reduced the blockage of xylem vessels by bacteria. The special peaks determined by FTIR further determined that GO attached to the xylem vessels at the base of the flower stem and its efficient water transport properties helped to adjust the water balance of cut flowers, preventing rapid drying. The oxidation-reduction state of GO on the stem incisions was determined by XPS analysis, proving that GO could enter the interior of the flower stem through the duct at the bottom of the stem, and when combined with the most effective preservative, “*Long Life*,” the anti-oxidation protection ability of GO was enhanced, further reducing the accumulation of ROS in cut flowers, thus delaying aging, although the detailed roles played by GO in the flower stem need further experimental proof.

## Data availability statement

The raw data supporting the conclusions of this article will be made available by the authors, without undue reservation.

## Author contributions

JZ contributed to conceptualization, supervision, writing – review and editing. YYW contributed to methodology, software, writing, visualization, investigation. YRW, SW, and XF helped visualization, investigation. YL, RZ, HH, and YZ helped data curation, software, and validation. All authors contributed to the article and approved the submitted version.
